# Lemmel Syndrome: A Rare Cause of Obstructive Jaundice

**DOI:** 10.7759/cureus.98012

**Published:** 2025-11-28

**Authors:** Idan Grossmann, Aubin Attila, Harshavardhan Sanekommu, Natasha Campbell, Lee Peng

**Affiliations:** 1 Department of Internal Medicine, Hackensack Meridian Health Jersey Shore University Medical Center, Neptune, USA; 2 Department of Gastroenterology and Hepatology, Hackensack Meridian Health Jersey Shore University Medical Center, Neptune, USA

**Keywords:** appropriate imaging, early identification and diagnosis, : lemmel syndrome, obstructive jaundice, periampullary duodenal diverticula

## Abstract

Obstructive jaundice is a potentially life-threatening condition with multiple etiologies. Lemmel syndrome is a rare and intriguing cause of obstructive jaundice, most commonly associated with a periampullary duodenal diverticulum. This syndrome presents a diagnostic challenge because its features are often subtle and can mimic more common causes of biliary obstruction. We report a case of a 68-year-old patient who presented with acute abdominal pain, had a broad differential, and was ultimately diagnosed with Lemmel syndrome. This case emphasizes the importance of recognizing the syndrome, as its presentation can overlap with several other conditions.

## Introduction

Obstructive jaundice is a serious condition that can be a medical emergency, most commonly caused by gallstones, malignancies, and strictures [1].

Lemmel syndrome is a rare cause of obstructive jaundice caused by compression of the common bile duct by a periampullary duodenal diverticulum, in the absence of choledocholithiasis or tumor [2]. Periampullary duodenal diverticula are anatomical anomalies found in approximately 27% of the general population. However, fewer than 1% of affected individuals develop Lemmel syndrome [3]. These diverticula typically occur within 2-3 cm of the ampulla of Vater and represent pouch-like outpouchings in the duodenum that can lead to biliary obstruction through direct compression or local inflammation [4]. Lemmel syndrome most commonly appears in elderly patients, with a male predominance, and clinically often presents with jaundice, mild or intermittent abdominal pain, and, in some cases, complications such as cholangitis or pancreatitis [5].

In this report, we present a 68-year-old male who developed acute abdominal pain and clinical signs consistent with obstructive jaundice. Initially, the presumed diagnosis favored more common etiologies; however, further workup revealed a different and much less common cause, prompting appropriate management. This case emphasizes the importance of maintaining a broad differential diagnosis to ensure timely treatment and prevent potential complications.

This case was previously presented as a poster at the American College of Gastroenterology (ACG) Annual Scientific Meeting on October 27, 2025 in Arizona, USA.

## Case presentation

A 68-year-old male with a significant medical history of hypertension, hyperlipidemia, type 1 diabetes mellitus, coronary artery disease, and chronic kidney disease (CKD) presented to the emergency department with an acute onset of epigastric abdominal pain, rated 8/10 in intensity, that had progressively worsened over the past two days. It was associated with nausea and vomiting, but no alleviating or aggravating factors were identified. The patient described the pain as constant and sharp in nature. He denied hematemesis, diarrhea, or recent travel history, and he denied any sick contacts.

On admission, the patient appeared in moderate distress due to abdominal pain. Vital signs were notable for a temperature of 99.1°F, blood pressure of 170/67 mmHg, and an oxygen saturation of 97% on room air. The physical examination was remarkable for scleral icterus and generalized abdominal tenderness without peritoneal signs.

Laboratory results revealed a bandemia of 14%, elevated glucose of 168 mg/dL, and an elevated total bilirubin of 2.1 mg/dL (direct bilirubin 1.8 mg/dL, indirect bilirubin 0.3 mg/dL). Liver enzymes were also abnormal, with an elevated alkaline phosphatase (173 U/L), AST (73 U/L), and GGT (225 U/L), while ALT was within the normal range (Table 1).

**Table 1:** Summary of laboratory results on admission BUN: Blood urea nitrogen; GGT: Gamma-glutamyl transferase; AST: Aspartate transaminase; ALT: Alanine transaminase

Parameter	Patient Value	Reference Range
WBC	5 ×10⁹/L	4.5–11.0 ×10⁹/L
Hemoglobin	12 g/dL	13.5–17.5 g/dL
Hematocrit	37%	41–53%
Platelets	320 × 10⁹/L	150–400 × 10⁹/L
Bands	14%	0–10%
Sodium	139 mmol/L	135–145 mmol/L
Potassium	3.8 mmol/L	3.5–5.1 mmol/L
Chloride	104 mmol/L	98–107 mmol/L
CO₂ (Bicarbonate)	27 mmol/L	22–29 mmol/L
BUN	26 mg/dL	7–20 mg/dL
Creatinine	1.39 mg/dL	0.7–1.3 mg/dL
Total Bilirubin	2.1 mg/dL	0.1–1.2 mg/dL
Direct (Conjugated) Bilirubin	1.8 mg/dL	0.0–0.3 mg/dL
Indirect (Unconjugated) Bilirubin	0.3 mg/dL	0.2–0.9 mg/dL
GGT	225 U/L	0–65 U/L
Alkaline Phosphatase	173 U/L	44–147 U/L
AST	73 U/L	10–40 U/L
ALT	46 U/L	7–56 U/L
Lipase	64 U/L	0–160 U/L

**Figure 1:**
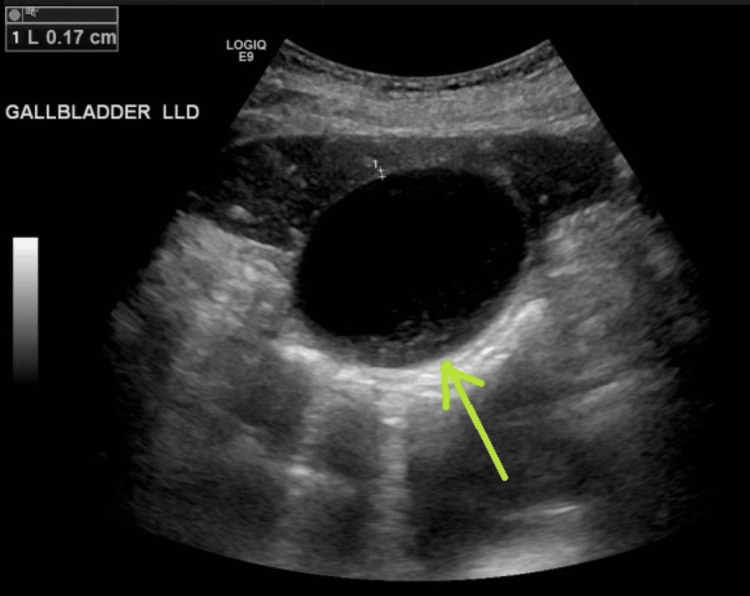
Right upper quadrant ultrasound demonstrating a distended gallbladder containing a small amount of echogenic sludge.

**Figure 2:**
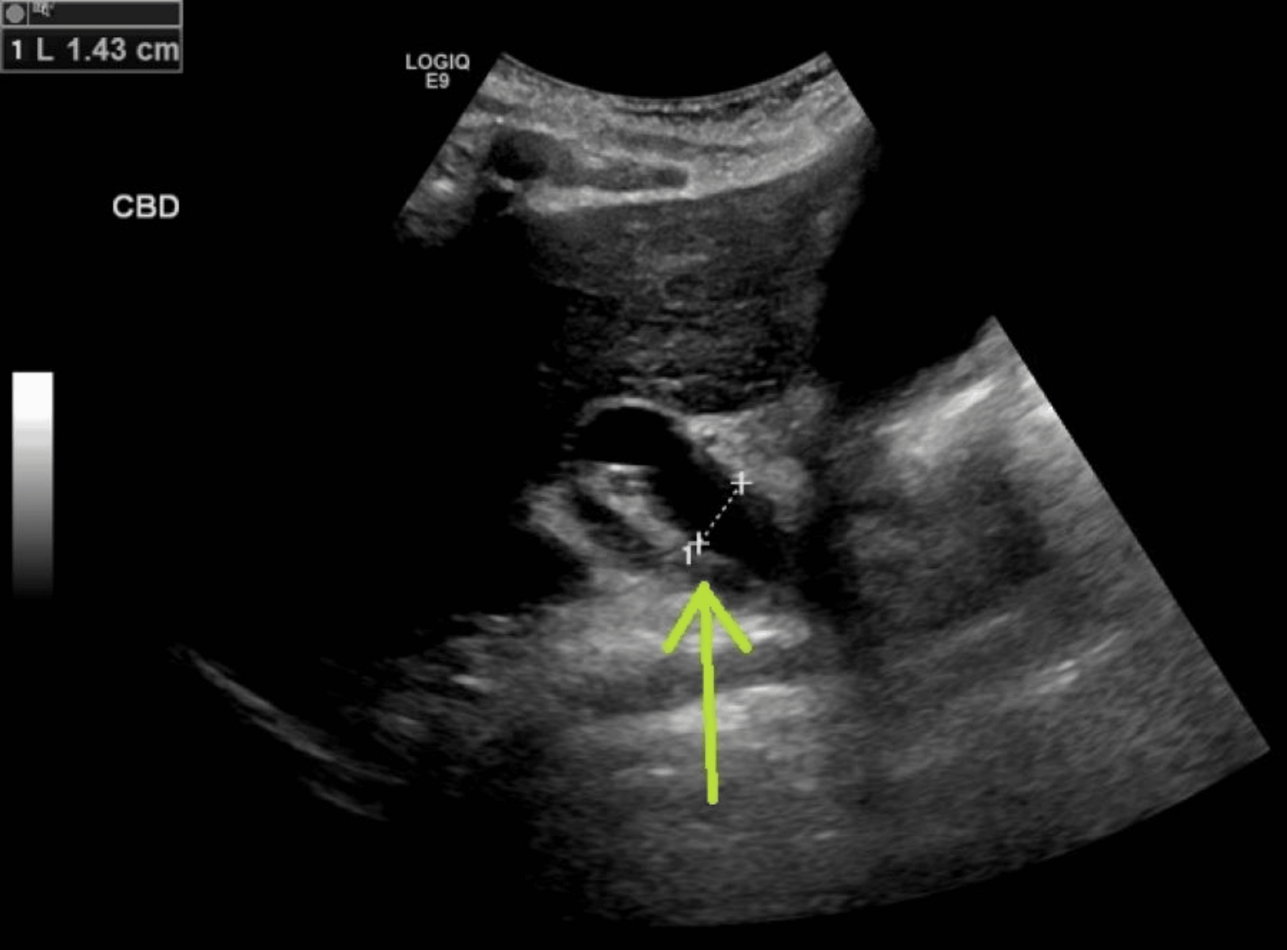
Right upper quadrant ultrasound demonstrating a dilated CBD.

**Figure 3:**
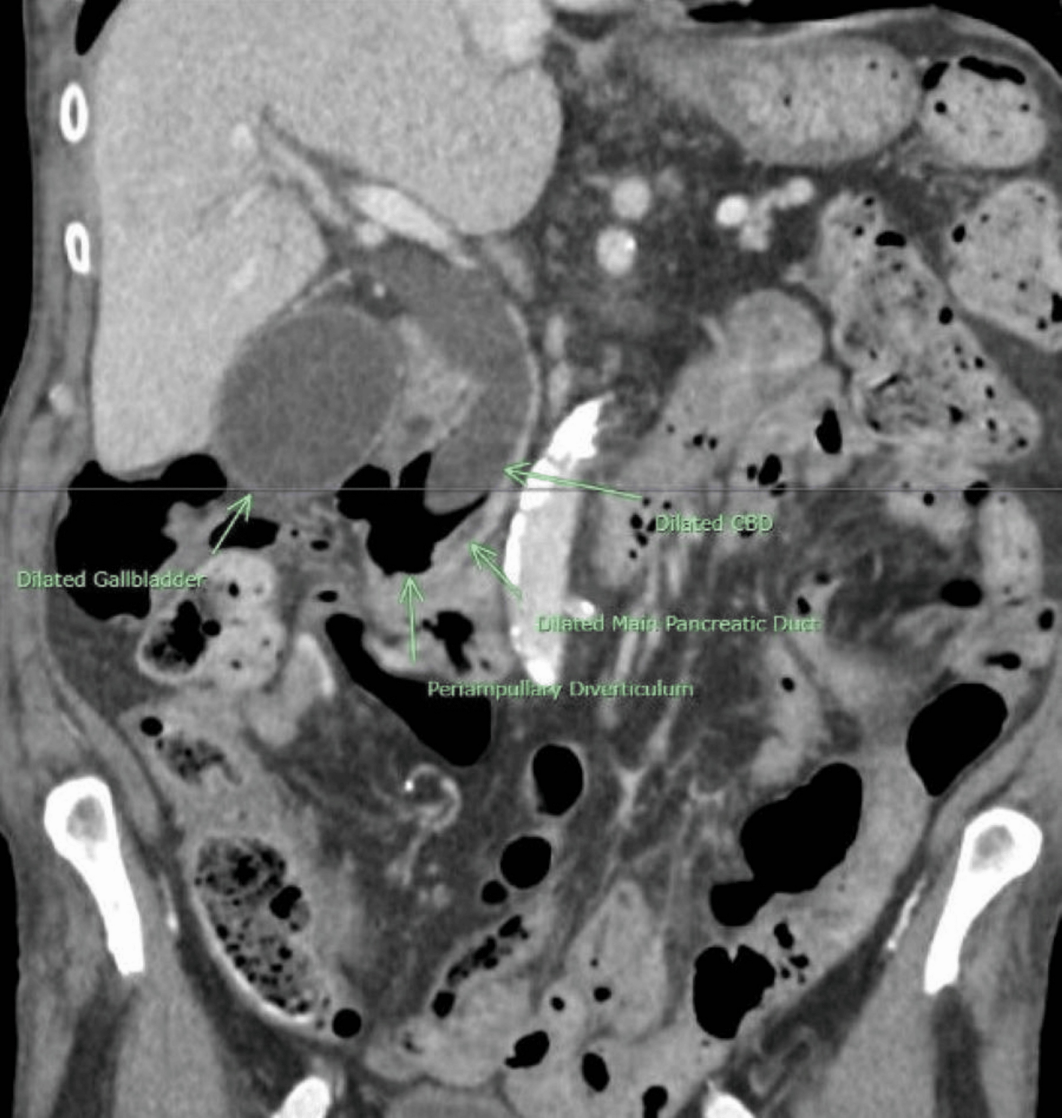
Coronal contrast-enhanced CT of the abdomen showing a periampullary diverticulum compressing the distal CBD with biliary and pancreatic ductal dilation.

**Figure 4:**
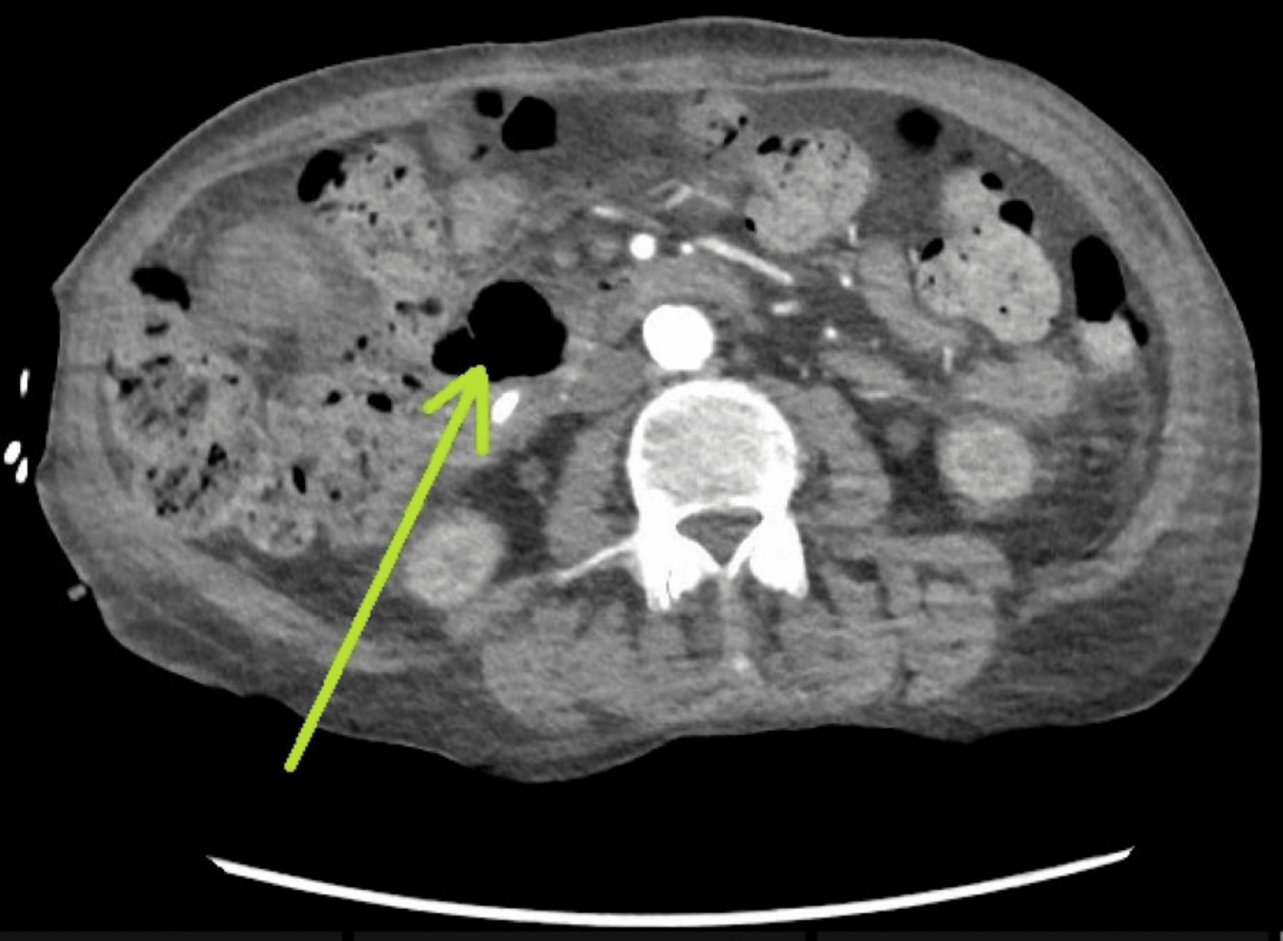
Axial contrast-enhanced CT of the abdomen showing a periampullary duodenal diverticulum compressing the distal common bile duct.

**Figure 5:**
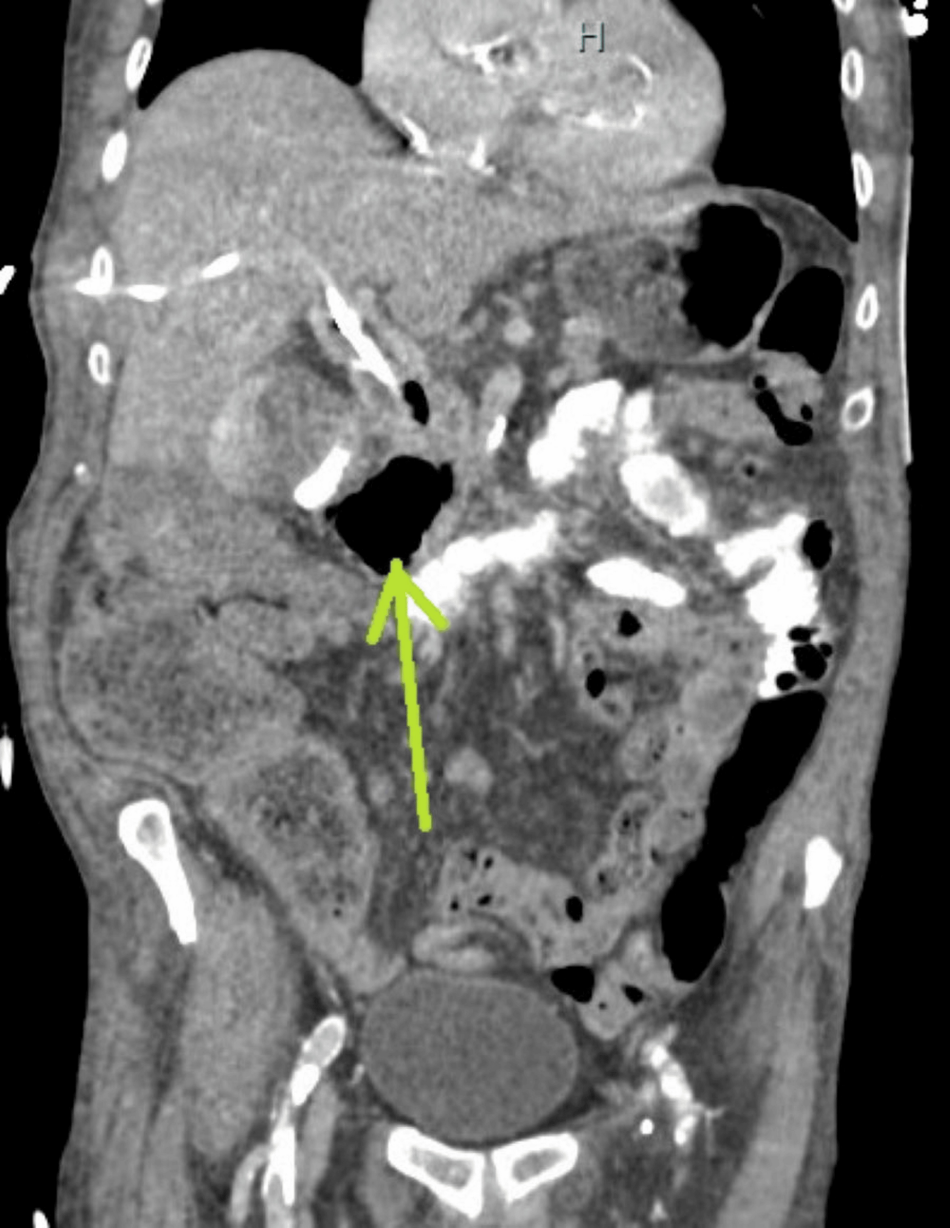
Another view of coronal contrast-enhanced CT of the abdomen showing a periampullary duodenal diverticulum compressing the distal common bile duct.

A right upper quadrant ultrasound revealed a distended gallbladder with sludge (Figure 1) and evidence of intrahepatic biliary dilation (Figure 2). A subsequent contrast-enhanced CT of the abdomen and pelvis demonstrated a distended gallbladder with moderate intrahepatic biliary and pancreatic dilation, as well as a dilated common bile duct (CBD) with an abrupt cut-off near the ampulla of Vater (Figure 3). Additionally, a small periampullary duodenal diverticulum was noted near the ampulla, located close to both the CBD and the pancreatic duct (Figures 4, 5). Magnetic resonance cholangiopancreatography (MRCP) confirmed the findings of a dilated CBD up to the level of the ampulla and a large periampullary duodenal diverticulum.

Given the patient's presentation and imaging findings, he was diagnosed with biliary obstruction likely secondary to the periampullary duodenal diverticulum. Broad-spectrum antibiotics were initiated empirically to cover possible cholangitis, based on the presence of jaundice, right upper quadrant abdominal pain, and laboratory evidence of cholestasis. However, the diverticulum’s location corresponded with the distal common bile duct cutoff, supporting it as the most likely cause of obstruction over other potential etiologies such as choledocholithiasis or tumor. An endoscopic retrograde cholangiopancreatography (ERCP) with stent placement was performed to relieve the biliary obstruction. The patient tolerated the procedure well. Within 24 hours, his abdominal pain decreased substantially, he began tolerating oral intake, and he no longer required analgesics. Laboratory values showed down-trending bilirubin and cholestatic enzymes, consistent with biochemical recovery. The patient was subsequently discharged in stable condition with outpatient follow-up for continued management of his biliary pathology and other comorbid conditions.

## Discussion

Lemmel syndrome is an uncommon cause of obstructive jaundice. Malignancy accounts for the majority of obstruction cases, comprising approximately 64%, while choledocholithiasis represents 24-33% [6]. In contrast, Lemmel syndrome develops only in a small minority of cases [3].

Lemmel syndrome results from periampullary duodenal diverticula, which are relatively common, with a reported prevalence of up to 27% in the general population based on endoscopic, radiographic, and autopsy studies. Clinically, these diverticula are frequently identified in elderly patients undergoing ERCP [4]. Despite their common occurrence, progression to Lemmel syndrome remains rare [7].

The clinical presentation of Lemmel syndrome is variable. The most common features include painless jaundice and mild, intermittent abdominal pain. Additional symptoms may develop secondary to complications of untreated Lemmel syndrome [5]. In our case, the patient presented with non-classical features, including acute and persistent abdominal pain, which added another layer of complexity to the diagnostic process. The presence of gallbladder sludge initially raised suspicion for gallstone-related obstruction, highlighting a common diagnostic pitfall. Moreover, periampullary diverticula can be subtle and are often difficult to interpret on standard imaging, potentially leading to misdiagnosis. Careful correlation of clinical findings with high-resolution cross-sectional imaging, as performed here with CT and MRCP, allowed accurate identification of the periampullary diverticulum as the source of biliary obstruction, thereby distinguishing Lemmel syndrome from gallstone-related disease.

The diagnosis of Lemmel syndrome relies primarily on imaging. In a recent series, endoscopic ultrasound (EUS) successfully identified all reported cases, whereas non-invasive modalities such as MRCP, CT, and contrast-enhanced abdominal MRI failed to establish the diagnosis [5]. In contrast, in our case, CT and MRCP were sufficient to confirm Lemmel syndrome. Early recognition has a significant impact on outcomes, and timely diagnosis is associated with excellent prognosis, while delayed or missed diagnosis may lead to complications such as cholangitis and pancreatitis [2, 3, 7, 8].

This case illustrates the rare and often diagnostically challenging presentation of Lemmel syndrome, which can easily be mistaken for more common causes of obstructive jaundice. It emphasizes the importance of maintaining a high index of suspicion and utilizing the most sensitive radiologic modalities when the diagnosis is considered, as well as the need for prompt treatment to achieve optimal outcomes.

## Conclusions

Lemmel syndrome, although uncommon, represents a clinically significant cause of obstructive jaundice in elderly patients without choledocholithiasis or malignancy. Its often nonspecific presentation and subtle periampullary diverticula on imaging pose diagnostic challenges, necessitating careful clinical assessment and targeted cross-sectional studies such as CT and MRCP. Timely recognition and appropriate endoscopic management facilitate rapid symptomatic and biochemical resolution while preventing complications, underscoring the need for clinicians to maintain a broad differential and vigilance for this rare entity.
